# A Case Report of a Large Goiter Resulting in Tracheal Deviation

**DOI:** 10.21980/J80645

**Published:** 2021-07-15

**Authors:** Thomas Powell, Geremiha Emerson

**Affiliations:** * The Ohio State University Wexner Medical Center, Department of Emergency Medicine, Columbus, OH

## Abstract

This is a case report of an extremely large mediastinal mass resulting in tracheal deviation in an 82-year-old female. Such a high degree of both goiter size and tracheal deviation is unusual in Western populations and highlights the potential dangers for patients with unknown mediastinal masses who may require intubation. Because this patient presented with altered mental status after a fall, intubation was considered for airway protection until a trauma chest x-ray revealed the previously unknown mass. Fortunately, this patient was able to protect her airway. In this report, we summarize the case and the latest literature about the dangers of intubation of patients with mediastinal masses. Paralyzing these patients often compromises their airways due to the loss of muscle tone which prevented the mass from obstructing the airway. Awake intubations and use of high ventilatory pressures are reviewed. Fortunately for this patient, her clinical course was unremarkable and she was discharged from the hospital in good condition with the decision not to have the mass removed. A history or a suggestion of a mediastinal mass should give any provider pause before securing the airway of a patient with this condition, and if possible, intubation should be performed using an awake technique.

**Topics:**

Mediastinal mass, goiter, image, x-ray, airway, thyroid mass.

**Figure f1-jetem-6-3-v4:**
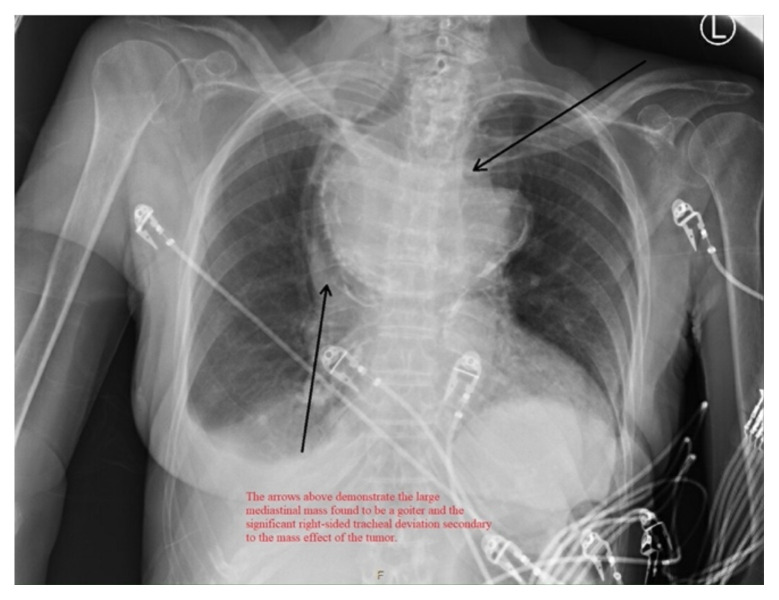


## Brief introduction

[Fig f1-jetem-6-3-v4]The patient is an 82-year-old female who presented to the emergency department as a trauma after a fall. Upon arrival to the department, she was found to be significantly altered with a Glasgow Coma Scale (GCS) of 9. A screening chest x-ray was performed, which revealed a large mediastinal mass with significant right-sided tracheal deviation. Fortunately for this patient, her oxygen saturation was within normal limits without use of supplemental oxygen or positive pressure. A CT Chest demonstrated this mass was a very large goiter extending from the thyroid to the anterior mediastinum. The patient was found to have pyelonephritis due to an ascending cystitis, from which she became altered and fell. Her mental status and function improved considerably after antibiotic therapy, and she was fit to be discharged from the hospital three days after presentation. She and her family, after discussion with the inpatient team, decided against surgical management of this large goiter opting for medical therapy instead. Unfortunately, this patient was then lost to clinical follow-up.

## Presenting concerns and clinical findings

Due to the very large mass, close evaluation of the patient’s airway status was maintained and the team elected to not intubate her for airway protection because the risk outweighed the benefit of the intervention. Despite her significant altered mental status, she was able to protect her airway until her mental status returned to baseline.

## Significant findings

In the image, one can see significant tracheal deviation around the right side of the mass (black arrows). This degree of deviation would make ventilation in a paralyzed patient extremely difficult, if not impossible.

## Discussion

Masses of the anterior mediastinum occur as one of typically four categories: thymomas, thyroid masses, lymphomas, and metastatic masses.^1^ Other large primary tumors such as squamous cell carcinomas can arise in this area and should be considered when encountering a large anterior mediastinal mass. As was the case in this patient, very large goiters represent 7–15% of anterior mediastinal masses^2^, with a malignancy rate reported as ranging from 3–15%.^3^

When treating a patient with respiratory failure of uncertain etiology, a mediastinal mass should be considered within the differential. Signs of severe obstruction include orthopnea, stridor, cyanosis, jugular venous distension, or even superior vena cava syndrome.^4^ Mediastinal masses often create great difficulty with maintaining airway patency, especially during general anesthesia. With the induction of general anesthesia, a loss of muscle tone during causes a collapse of the mass onto the bronchial tree. This creates an obstruction of the airway and requires large amounts of airway pressure to overcome; pressures as high as 50cmH_2_O may be required to ventilate these patients. 1 If one has to intubate these patients, great care must be taken to perform an awake fiberoptic intubation in either the upright or prone positioning to allow for the best airway protection possible. 5 The patient should not be paralyzed if at all possible due to potential mass collapse on the airway. 6 Performing a mainstem intubation is also a consideration to maximize oxygenation in one lung alone. If fiberoptic bronchoscopy fails, rigid bronchoscopy, usually performed by an otolaryngologist or pulmonologist, can be attempted. 1 Case reports of other devices such as a double-lumen endotracheal tube or placing two 6.0 endotracheal tubes into the bronchi under fiber-optic assistance have been successfully demonstrated.^7^,^8^ One source goes so far as to propose placing these patients on extracorporeal membrane oxygenation in-lieu of traditional oxygenation. 1

Mediastinal masses are an uncommon but potentially catastrophic cause of ventilatory failure. It is important to consider this diagnosis in your differential when confronted by a patient with dyspnea and/or stridor, especially in patients with unclear medical history. If a mediastinal mass is suspected, a chest x-ray prior to considering intubation should be obtained to ascertain the size of the mass and if it has an associated tracheal deviation. If found, awake fiberoptic intubation is the preferred method to maintain the patient’s intrinsic respiratory drive while airway access is obtained. Once intubated, the physician and respiratory therapist caring for the patient must be ready to apply very high inspiratory pressures to ventilate through the mass compressing the airway. Although not airway compromising in this patient, the extensive distortion of the lower airway in this patient should give providers pause when confronted with a compromised airway in patients with newly discovered mediastinal masses. As with any airway, a well thought out and communicated plan, with adjunctive therapies on standby, will greatly increase ventilatory success in patients with both known and previously unknown mediastinal masses.

## Supplementary Information




