# Supplementary figures and images for: A Case Report of a Large Goiter Resulting in Tracheal Deviation
Source: J Educ Teach Emerg Med. 2021 Jul 15;6(3):V4–6. doi: 10.21980/J80645 (PMC10332692; doi:10.21980/J80645)

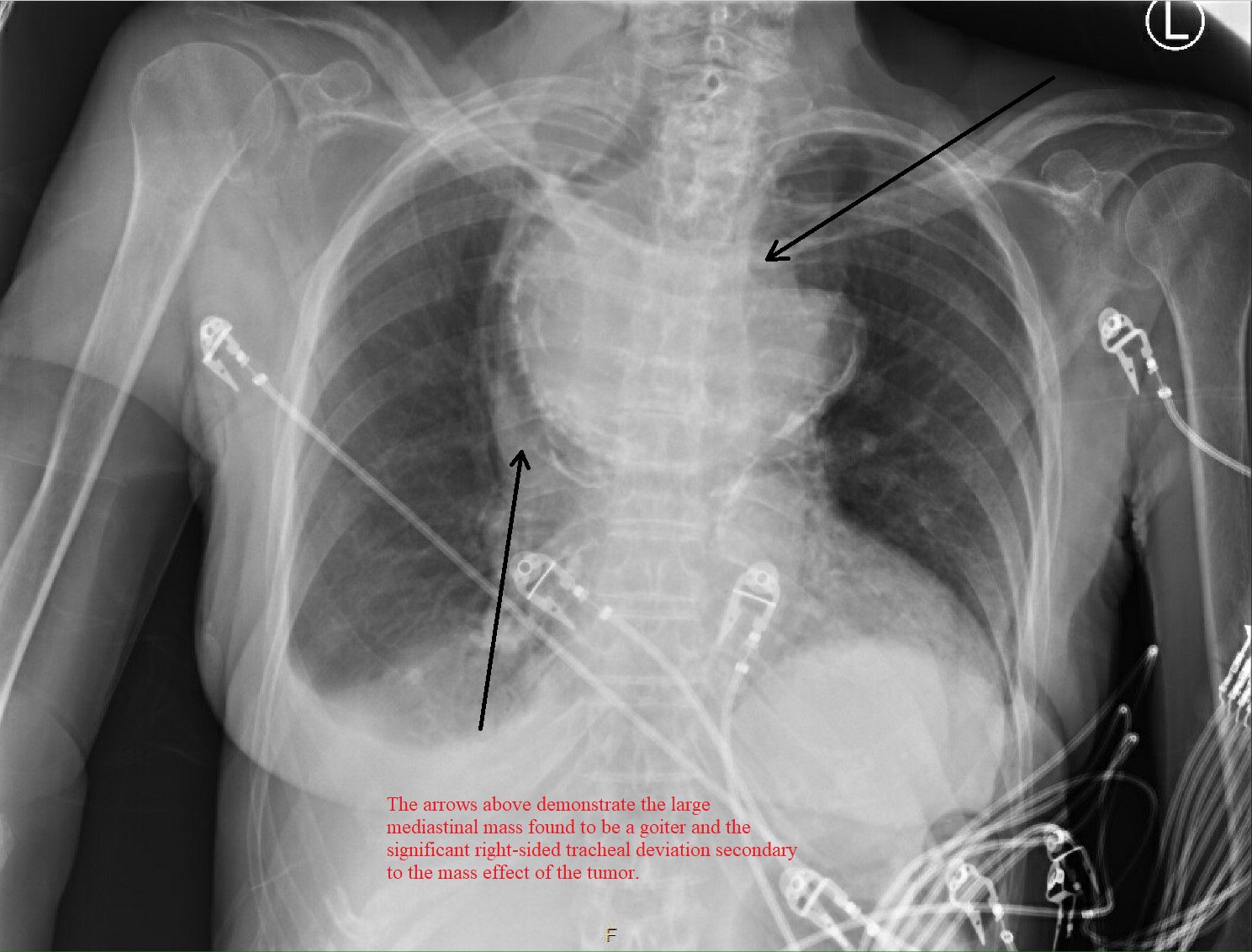

Supplement: Supplementary file 1 [file jetem-6-3-v4-supp1.jpg]

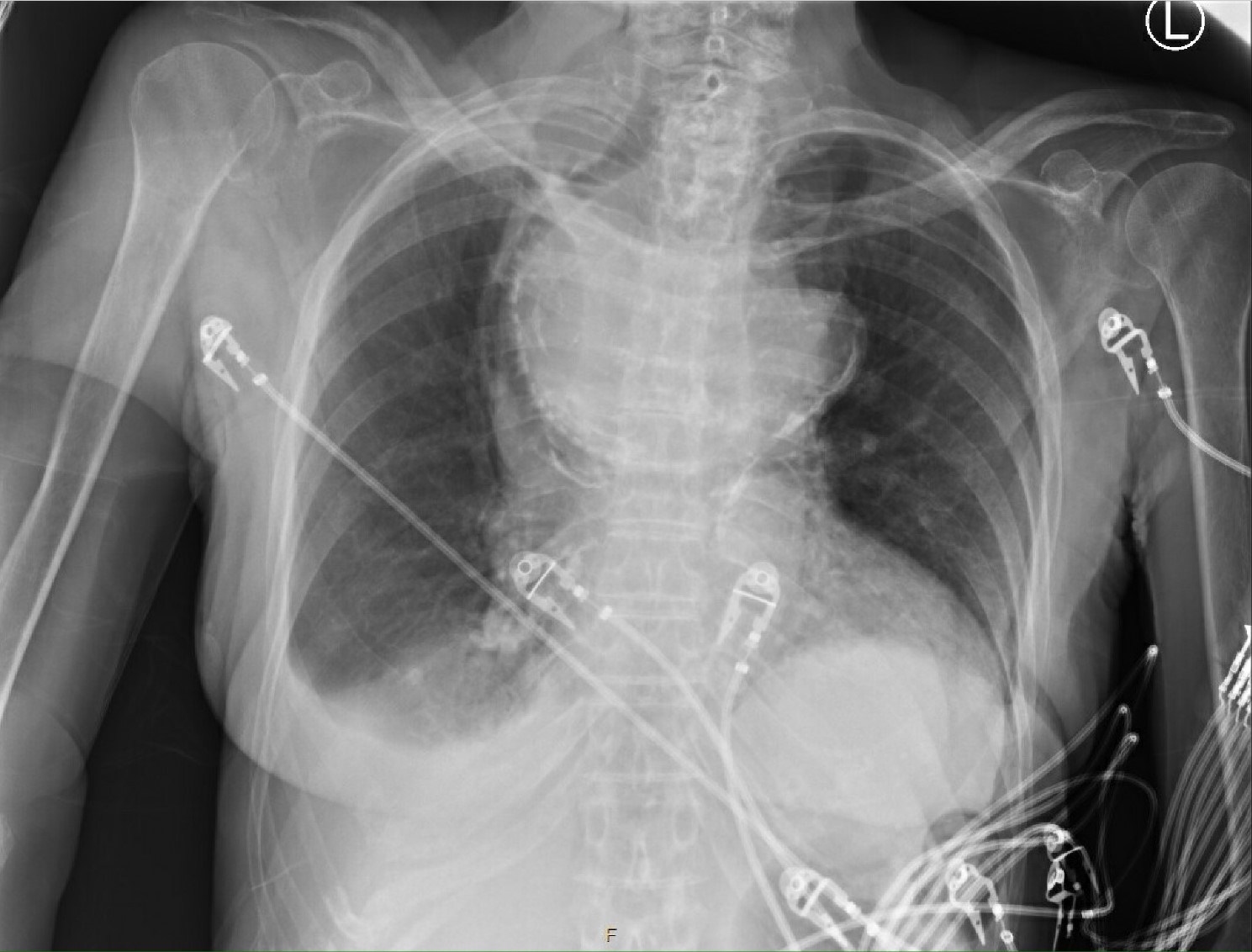

Supplement: Supplementary file 2 [file jetem-6-3-v4-supp2.jpg]
